# Impact of Elevated C-Reactive Protein on Survival Outcomes of Patients with Small Renal Masses: A Retrospective Multicenter Analysis

**DOI:** 10.3390/curroncol33060327

**Published:** 2026-06-01

**Authors:** Margaret F. Meagher, Natalie Birouty, Giacomo Musso, Dattatraya Patil, Kazutaka Saito, Yosuke Yasuda, Dhruv Puri, Benjamin Baker, Kit Yuen, Jacob L. Roberts, Aaron Ahdoot, Omer Baker, Mai Dabbas, Julian Cortes, Yasuhisa Fujii, Viraj Master, Michael Liss, Ithaar H. Derweesh

**Affiliations:** 1 Department of Urology, University of California San Diego, San Diego, CA 92103, USA; 2 Department of Urology, Emory University School of Medicine, Atlanta, GA 30322, USA; 3 Department of Urology, Tokyo Medical and Dental University, Tokyo 113-8519, Japan; 4 Department of Urology, Rush University Medical Center, Chicago, IL 60612, USA

**Keywords:** algorithm, carcinoma, renal cell, C-reactive protein, nephrectomy, survival analysis

## Abstract

**Objective:** This study aimed to investigate the impact of elevated CRP on survival outcomes in small renal masses (SRM). **Methods:** This was a multi-institutional retrospective analysis of SRM (≤3 cm) managed surgically. The cohort was divided into elevated CRP (≥0.5 mg/dL) vs. non-elevated CRP groups (<0.5). The primary outcome was all-cause mortality (ACM). The secondary outcomes were non-cancer (NCM) and cancer-specific mortality (CSM). Cox-regression analysis was used to elucidate predictive factors for mortality outcomes. Kaplan–Meier Analysis (KMA) was performed to analyze 10-year overall (OS), cancer-specific (CSS) and non-cancer-specific survival (NCS). **Results:** A total of 1001 patients were analyzed (309 non-elevated CRP/692 elevated CRP; median follow-up 70 months). Elevated CRP was an independent predictor for ACM (HR = 2.60, *p* < 0.001) NCM (HR = 2.90, *p* = 0.002), and CSM (HR = 1.20, *p* = 0.011). KMA comparing elevated vs. non-elevated groups revealed greater 10-year OS (*p* < 0.001) and NCS (*p* < 0.001) for non-elevated CRP, but no significant difference in 10-year CSS (*p* = 0.295). A total of 83 deaths were observed in elevated CRP (71 NCM/12 CSM—all clear-cell histology). The sensitivity/specificity of elevated CRP was 0.87/0.33, 0.75/0.81, and 0.90/0.33 for ACM, CSM, and NCM. By utilizing CRP for a decision-making algorithm prioritizing biopsy in CRP elevation and offering surveillance in benign or indolent histology, surgery may be avoided in 218 patients, in whom there were 38 fatalities, all NCM. **Conclusions:** Elevated CRP was an independent predictor of survival outcomes in SRM ≤ 3 cm. From a competing mortality standpoint, patients with elevated CRP had significantly worsened NCM compared to CSM. In such patients, upfront oncologic risk stratification through biopsy may be considered, and indolent/low-grade neoplasms should be strongly considered for non-surgical management.

## Simple Summary

The optimal management of small renal masses (SRMs, <4 cm) must balance the need for adequate cancer control with the risk of overtreatment. Current guidelines recommend consideration of serial imaging as an initial management strategy for patients with SRM, with surgical excision reserved for growing masses in young, healthy patients. However, even with increased de-emphasis on immediate surgery, overtreatment of benign and indolent renal cancer remains a quality-care concern. CRP is an inflammatory marker that is elevated in a variety of disease states such as cancer. We sought to investigate the prognostic value of pre-operative CRP in predicting survival outcomes in patients with small renal masses. We hope to provide additional information to guide patient counseling, with the goal of offering surveillance and surgery to appropriate patients while taking into account the competing risks of mortality.

## 1. Introduction

The management of small renal masses (SRMs) represents an important therapeutic dilemma [1]. Defined as an incidentally found mass less than or equal to 4 cm, SRMs have increased in incidence over the past several decades due to expanded utilization of cross-sectional imaging [2]. Optimal management of SRMs must balance the need for oncologic control against the risk of overtreatment. [3]. Current guidelines recommend consideration of active surveillance as an initial management strategy for patients with tumors less than 3 cm, even for young and healthy patients, while partial nephrectomy is the reference standard for surgical excision [1,4]. Despite an increased understanding of the heterogeneous potential of small masses and an increased emphasis on organ-conserving and surveillance strategies, overtreatment of benign and indolent renal caner continues to be a quality-of-care concern [5,6].

Renal cell carcinoma is a metabolically active malignancy in which inflammatory markers have emerged as potential pathophysiologic mediators [7]. C-reactive protein (CRP) is a an acute-phase reactant and a marker of systemic inflammation that increases in response to a variety of disease states including cardiovascular dysfunction, infection, rheumatologic conditions, trauma and cancer [8]. Emerging reports have suggested an association between elevated CRP and high-grade small renal masses [9]. While previous groups have explored various biomarkers such as urine methylation for SRM stratification, CRP is an ideal biomarker as it is both widely available and financially benign [10]. We sought to investigate the impact of elevated CRP on survival outcomes in patients with renal mass sizes ≤ 3 cm.

## 2. Patients and Methods

### 2.1. Patient Population

This was a retrospective, international multi-institutional analysis utilizing the INMARC (INternational Marker Consortium for Renal Cancer) registry. The INMARC registry is not a public dataset, and data shared between institutions with data sharing agreements are not accessible to investigators not part of the consortium due to IRB limitations. Participating institutions included the University of California, San Diego; Emory University; Tokyo Medical and Dental University; and Rush University. Included patients underwent surgical extirpation (radical or partial nephrectomy) between 1/2006 and 1/2020 for small renal masses (clinical size ≤ 3 cm). We chose the 3 cm threshold for analysis as it is the size threshold for consideration of active surveillance in AUA guidelines. [1]. The study was conducted in accordance with the Declaration of Helsinki and approved by the Institutional Review Board of UC San Diego (#151138, approved 30 April 2024). Institutional Review Board approval was additionally obtained at all participating institutions. We excluded patients presenting with tumor sizes > 3 cm, nodal or distant metastatic disease, or incomplete records.

### 2.2. Data Collection

The data were de-identified and entered into institutional datasets by database managers after surgery [7,11]. The collected variables included demographic and clinical disease data at time of diagnosis [age, sex, body mass index (BMI)], hypertension (HTN), diabetes mellitus (DM), coronary artery disease (CAD), C-reactive protein (CRP). Treatment data (surgical method and approach) was collected with operative variables [estimated blood loss (mL) and 30-day complications (Clavien-Dindo) [12]. CRP was measured in mg/dL and collected within 30 days prior to surgery. Survival outcomes, including recurrence-free survival, non-cancer-specific survival and overall survival at last follow-up were recorded. Cause of death was ascertained by treating urologic oncologists at each participating institution. Cancer-specific mortality was recorded if the cause of death was deemed to be related to RCC, while non-cancer-specific mortality was recorded if the cause of death was deemed to be unrelated to RCC.

### 2.3. Data Analysis

The cohort was divided into elevated CRP (greater than or equal to 0.5 mg/dL) vs. non-elevated CRP (less than 0.5 mg/dL) groups for the analyses [7]. A threshold of >0.5 mg/dL was chosen for elevated CRP as this corresponds with our institutional assays [7]. The primary outcome was all-cause mortality (ACM)/overall survival (OS) measured from date of diagnosis to date of last follow-up. The secondary outcomes were cancer-specific mortality (CSM)/cancer-specific survival (CSS), non-cancer mortality (NCM)/non-cancer-specific survival (NCS), and recurrence/recurrence-free survival (RFS). Descriptive analyses were conducted utilizing Student’s *t*-test/ANOVA and Fisher’s exact test for continuous and categorical variables, respectively. Normality of continuous variables was assessed via histogram. Cox regression analysis was utilized to elucidate predictive factors for ACM, CSM, NCM, and progression. We performed a stepwise backward model selection, with exclusion of factors with *p* > 0.2 on univariate regression analysis. The proportionality of hazards was verified via log-minus-log plots. Kaplan–Meier Analysis (KMA) was performed to analyze comparative analyses of survival for elevated CRP and non-elevated CRP groups, comparing 10-year NCS and CSS for each group via log-rank test.

## 3. Results

A total of 1001 patients were analyzed (309 non-elevated CRP, 692 elevated CRP). Median follow-up for the cohort was 67 months. Table 1 demonstrates demographic variables and clinical disease characteristics. The elevated CRP group had a significantly greater proportion of diabetes (8.4% vs. 18.1%, *p* < 0.001), hypertension (19.4% vs. 32.4%, *p* < 0.001), CAD (7.8% vs. 15.1%, *p* < 0.001), and had a higher BMI at diagnosis (22.7 vs. 26.2, *p* < 0.001). Groups did not vary significantly with respect to age at diagnosis (*p* = 0.239) or clinical tumor size (*p* = 0.535). There was a higher percentage of open surgery performed in the elevated CRP group (19.4% vs. 4.9%, *p* < 0.001). There was no difference with respect to surgical method (*p* = 0.252), EBL (*p* = 0.180), or 30-day complications (*p* = 0.450).

Table 2 displays Cox regression multivariable analyses for factors associated with survival outcomes. Cox regression revealed increasing age (HR 1.04, *p* < 0.001), DM (HR 1.64, *p* = 0.041), increasing tumor size (HR 1.47, *p* = 0.017), and elevated CRP (HR 2.60, *p* < 0.001) to be independent risk factors for ACM. Increasing age (HR 1.05, *p* < 0.001), increasing tumor size (HR 1.47, *p* = 0.017), and elevated CRP (HR 2.90, *p* = 0.002) were also independently associated with worsened NCM. Elevated CRP was independently associated with worsened CSM (HR 1.20, *p* = 0.011).

**Figure 1:**
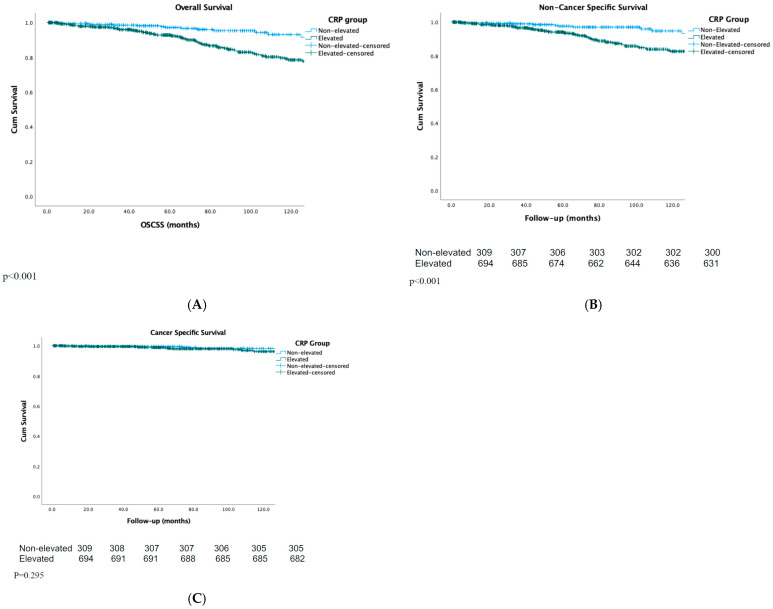
Kaplan–Meier Analyses for survival outcomes comparing elevated vs. non-elevated CRP groups for OS (**A**), NCS (**b**), and CSS (**C**).

Figure 1 displays the 10-year Kaplan–Meier analyses for OS, NCS, and CSS. Comparing non-elevated vs. elevated CRP groups, KMA revealed significantly higher 10-year OS (non-elevated = 91% vs. elevated = 79%, *p* < 0.001) and 10-year NCS (non-elevated = 92% vs. elevated = 83%, *p* < 0.001). On the other hand, no significant difference was noted between the groups with respect to 10-year CSS (non-elevated = 97%, elevated = 96%, *p* = 0.295).

**Figure 2:**
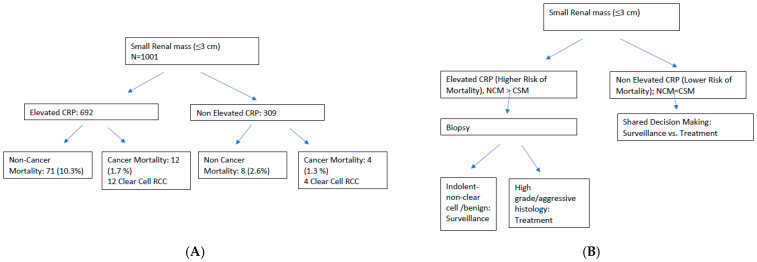
(**A**) Flow diagram of outcomes of SRM stratified by CRP status. (**B**) Decision-making tree based on CRP risk stratification.

Figure 2A delineates outcomes for patients by CRP elevation status. In the elevated CRP cohort, 71 (10.3%) had a non-cancer mortality event, while 12 (1.7%) experienced cancer-specific mortality. Overall, 100% of cancer-specific mortality was in patients with clear-cell histology. In the non-elevated CRP cohort, 8 (2.5%) patients experienced non-cancer mortality, while 4 (1.3%) experienced cancer-specific mortality; 100% of cancer-specific mortality was in patients with clear-cell histology. Using the above values, we calculated a sensitivity and specificity of 0.87 and 0.33, 0.75 and 0.81, and 0.90 and 0.33 for ACM, CSM, and NCM, respectively.

Figure 2B displays our proposed decision-making algorithm. In patients with elevated CRP, we found that non-cancer mortality events were more frequent than cancer-specific mortality events. In this setting, we recommend proceeding with biopsy to stratify risk. In patients with indolent histology (benign neoplasms/papillary RCC/chromophobe RCC), we recommend proceeding with surveillance as there are competing sources of non-cancer mortality. In patients with high-grade or aggressive histology (clear-cell RCC/variant histology), we recommend consideration of treatment, as cancer-specific mortality risk is higher than non-cancer mortality. In patients with non-elevated CRP, we found that non-cancer mortality and cancer-specific mortality were relatively equivalent. In this setting, we recommend shared decision-making between patient and physician regarding active surveillance versus treatment. If such a strategy was employed using CRP as a gate-keeper to biopsy and with surveillance for non-clear-cell/variant histology tumors, upfront surgery could have been avoided in up to 218 patients, in whom there were 38 fatalities, all of which were non-cancer related.

## 4. Discussion

We evaluated a robust cohort of patients with renal mass sizes ≤ 3 cm with a 70-month median follow-up. In this cohort with long-term follow-up, we noted elevated CRP to be a significant predictor of survival outcomes in renal masses ≤ 3 cm. CRP elevation was associated with worsened overall survival and non-cancer mortality in SRM, while its impact on cancer-specific mortality was less pronounced. For patients with elevated CRP, renal mass biopsy may be considered to guide management strategy whereupon biopsies yielding aggressive histology (high-grade disease, variant histology) should be considered for treatment given the risk of CSM in this group, while patients with low-risk histology (low-grade clear or papillary cell/chromophobe/oncocytic neoplasms) can be surveilled given the overall higher risk of NCM. On the other hand, patients with normal CRP have similar NCS and CSS rates at the 5- and 10-year mark and may be successfully managed based on clinical criteria and shared decision-making as regards intervention versus surveillance. These findings are novel and call for conformation by other cohorts but otherwise represent the first attempt to rationally employ biopsy by use of a prognostic biomarker which enhances comparative mortality analysis and creates specific recommendations for surveillance of indolent cancer histologies in addition to benign tumors. Taken together, application of a CRP-based risk stratification protocol may further reduce overtreatment of small renal masses by extending the surveillance rubric to low-risk malignancy while preserving option for intervention in high-risk tumor histologies [13,14].

In recent years, CRP has gained increased attention as a biomarker for RCC [7,15,16]. In the metastatic setting, Stares et al. prospectively followed 160 patients over an average of 50 months and found that CRP was independently predictive of time to systemic therapy (HR 2.47, *p* < 0.001), with those with CRP >10 mg/L receiving therapy at 13.8 months versus 55.1 months for those with CRP <10 mg/L (*p* = 0.001) [17]. Steffens et al., in an analysis of 1161 patients who underwent surgical extirpation for stage I–IV disease, found that CRP > 10 mg/L was independently associated with both CSS (2.58, *p* <0.001) and OS (HR 2.48, *p* < 0.001) [12]. Similarly, previous work from our group demonstrated that elevated CRP ( >5 mg/L) was associated with worsened all-cause mortality (HR 4.13, *p* < 0.001), with differential survival persisting across AJCC stages [7]. A direct comparison of our results to the aforementioned studies is challenging as we included both benign and malignant histology. To our knowledge, however, ours is the first study to propose inclusion of CRP as an element of a decision tree for electing surveillance versus treatment in cortical renal neoplasms.

The historic role of renal mass biopsy has been controversial given concerns that low diagnostic yield may not outweigh potential risks, such as tumor seeding and renal hemorrhage [18,19,20]. Current guidelines suggest that renal mass biopsy has high accuracy but do acknowledge the risks inherent to biopsy and, as such, only recommend biopsy if results will influence management [1,21]. Colakerol et al. conducted an analysis of 71 patients in order to define the predictive role of CRP on the diagnostic outcome of renal mass biopsy and found that elevated CRP (>10 mg/L) had a sensitivity of 76.6% and specificity of 81.8% for malignant histology [22]. Our study takes these findings a step further by examining the oncologic potential of malignant masses in patients with elevated CRP. In our cohort of ≤3 cm SRM patients, we noted that all-cancer mortalities were due to clear-cell histology. Given this finding, we propose that patients with elevated CRP but low-risk, non-clear-cell histology can be safely surveilled given the greater risk of competing mortality from non-oncologic causes.

Halvorsen et al. proposed an algorithm to guide management decisions based on biopsy results by incorporating histology, clinical tumor size, performance status, and depth of the mass on biopsy to create a risk-stratified decision tree [23]. The authors analyzed a cohort of 151 patients with renal masses ≤ 4 cm who underwent biopsy. Biopsy revealed cell type and grade in 133 patients, allowing the hypothetical assignment of surveillance vs. treatment using an algorithm incorporating small renal mass size and histological risk group. Biopsy called for surveillance of 36 small renal masses and treatment of 97 small renal masses. Final pathology showed 11 patients initially assigned to surveillance should have been assigned to treatment (8.3% of all patients, 31% of those recommended for surveillance), whereas no patients moved from treatment to surveillance. Agreement between biopsy and final pathology was 92%. Using management based on final pathology as the reference standard, biopsy had a negative predictive value of 0.69 and positive predictive value 1.0 for determining management. Rahbar et al. applied a modified version of this algorithm to 1175 patients who underwent robotic partial nephrectomy and found that 52% of patients in the active surveillance cohort had safe treatment [24].

By incorporating information regarding survival outcomes, our algorithm attempts to weigh data regarding competing sources of mortality and, for the first time, adds a biomarker CRP to the decision tree to proceed with biopsy. This decision tree has a sensitivity of 0.87 for all-cause mortality, 0.75 for cancer-specific mortality, and 0.90 for non-cancer mortality. We hypothesize that incorporation of CRP into clinical decision-making algorithms will assist clinicians in prioritizing rational and cost-effective risk stratification by preferentially directing biopsy to patients at high risk for mortality, reducing overtreatment and offering treatment for patients with high-risk small masses due to histology, who may otherwise be missed by algorithms which rely on tumor size as the only threshold for surveillance or definitive management [25,26]. On the other hand, the low specificities noted for ACM (0.33) and NCM (0.33) in the setting of high specificity for CSM (0.81) support the utility of CRP as a clinical decision-making tool in addition to biopsy for risk stratification.

As a retrospective, multicenter analysis, our study is limited by the inherent biases attributed to this study design. Additionally, we do not have a surveillance cohort and thus our results require validation. Importantly, while our analytic cohort has the histology to correlate with mortality outcomes and most active surveillance cohorts have a very small proportion of patients with biopsy-proven histology, our proposed algorithm is limited by the assumption that all masses which may have an indication for biopsy are amenable to biopsy, and that biopsy has a 100% accuracy rate. While both assumptions are optimistic, most contemporary cohorts report accuracy rates for histology with biopsy that well surpass 90% [27]. It should be noted that CRP may be elevated in a variety of disease states which also contribute to mortality. Furthermore, while CRP may not represent the ‘holy grail’ of a sensitive and specific marker for RCC, a growing body of literature supports its utilization in RCC risk stratification, and CRP measurements are part of the Japanese Guidelines on RCC [28]. Furthermore, CRP is a cost-effective and widely available test, as well as an established tool for managing cardiovascular and infectious diseases [29,30].

## 5. Conclusions

In addition to tumor size, elevated CRP was an independent predictor of survival outcomes in SRM ≤ 3 cm. From a competing mortality standpoint, patients with elevated CRP had significantly worsened ACM and NCM compared to CSM. In such patients, oncologic risk stratification through biopsy may be considered, offering surveillance to patients with benign or indolent neoplasms or a high burden of comorbidities, while proceeding with treatment in patients with aggressive histology.

## Figures and Tables

**Table 1:** Demographics, clinical disease characteristics, surgical characteristics.

Variable	Non-Elevated (*n* = 309)	Elevated (*n* = 692)	*p*-Value
Mean Age (years, IQR)	59.0 (48–66)	59.0 (50–67)	0.239
Sex (*n*, %)			0.063
Female	197 (65.8)	440 (63.6)	
Male	112 (36.2)	252 (46.4)	
Median BMI (kg/m^2^, IQR)	22.70 (20.87–24.84)	26.20 (23.06–30.90)	<0.001
CAD (*n*, %)	24 (7.8)	105 (15.1)	<0.001
HTN (*n*, %)	60 (19.42)	224 (32.37)	<0.001
DM (*n*, %)	26 (8.41)	125 (18.10)	<0.001
ECOG (*n*, %)			<0.001
0/1	257 (83.2)	270 (39.0)	
2+	52 (16.8)	422 (61.0)	
Mean Clin. Tumor Size (cm, SD)	2.1 (1.6–2.6)	2.1 (1.8–2.5)	0.535
Surgical Method (*n*, %)			0.252
Partial	231 (74.8)	492 (71.1)	
Radical	78 (25.2)	200 (28.9)	
Surgical Approach (*n*, %)			<0.001
Open	18 (5.8)	133 (19.2)	
Minimally Invasive	291 (94.2)	559 (80.8)	
Median EBL (mL, IQR)	163.5 (74.75–275)	150 (70–318.5)	0.18
30-Day Complication (*n*, %)	24 (7.7)	68 (9.8)	0.45
Mean Path Tumor Size (cm)	2.07 (0.88)	2.26 (1.57)	0.007
Histology (*n*, %)			<0.001
Clear	219 (70.9)	476 (68)	
Papillary	20 (6.4)	98 (14.1)	
Chromophobe	30 (9.7)	42 (6.1)	
Variant	17 (5.5)	53 (7.7)	
Benign	23 (7.4)	20 (2.9)	
Grade (*n*, %)			0.014
Low	234 (79%)	465 (71.3)	
High	62 (21%)	187 (28.7)	
ACM (*n*, %)	12 (3.9)	83 (12.0)	<0.001
NCM (*n*, %)	8 (2.6)	71 (10.3)	<0.001
CSM (*n*, %)	4 (1.3)	12 (1.7)	0.607

**Table 2:** Multivariable analyses.

**All-Cause 95.**	**Multivariable Analysis**		
**Variable**	**HR**	**95% CI**	***p*-value**
Increasing age (continuous)	1.04	1.02–1.06	**<0.001**
Sex (male vs. female)	1.22	0.80–1.86	0.368
Increasing BMI (continuous)	0.99	0.95–1.03	0.747
CAD (yes vs. no)	1.21	0.69–2.13	0.501
HTN (yes vs. no)	1.40	0.85–2.33	0.189
DM (yes vs. no)	1.64	1.02–2.63	**0.041**
Increasing tumor size (cm)	1.47	1.07–2.00	**0.017**
CRP elevation (yes vs. no)	2.60	1.49–4.51	**<0.001**
**Non-Cancer Mortality**	**Multivariable Analysis**		
**Variable**	**HR**		
Increasing age (continuous)	1.05	1.03–1.07	**<0.001**
Sex (male vs. female)	1.03	0.65–1.64	0.898
Increasing BMI (continuous)	0.99	0.95–1.04	0.704
CAD (yes vs. no)	1.21	0.72–2.48	0.359
HTN (yes vs. no)	1.40	0.89–2.46	0.249
DM (yes vs. no)	1.64	0.98–2.89	0.583
Increasing tumor size (cm)	1.47	1.08–2.17	**0.017**
CRP elevation (yes vs. no)	2.90	1.51–5.60	**0.002**
**Cancer-Specific Mortality**	**Multivariable Analysis**		
**Variable**	**HR**		
Increasing age (continuous)	1.01	0.99–1.07	0.128
Histology (versus clear)	1.07	0.87–1.11	0.938
High grade (yes vs. no)	1.01	0.92–1.05	0.987
Increasing tumor size (cm)	1.02	0.88–1.07	0.980
CRP elevation (yes vs. no)	1.20	1.04–1.57	**0.011**

## Data Availability

The data contained in the registry is not IRB-approved to be shared publicly, and cannot be made available.
